# Advanced AI-driven approach for enhanced brain tumor detection from MRI images utilizing EfficientNetB2 with equalization and homomorphic filtering

**DOI:** 10.1186/s12911-024-02519-x

**Published:** 2024-04-30

**Authors:** A. M. J. Zubair Rahman, Muskan Gupta, S. Aarathi, T. R. Mahesh, V. Vinoth Kumar, S. Yogesh Kumaran, Suresh Guluwadi

**Affiliations:** 1 Al-Ameen Engineering College (Autonomous), Karundevanpalayam, Nanjai Uthukuli (P.O), Erode, 638104 Tamil Nadu India; 2 Department of Computer Science & Engineering, Faculty of Engineering and Technology, JAIN (Deemed-to-be University), Bengaluru, 562112 India; 3 Department of CSE (AI & ML), Ramaiah Institute of technology, Bangalore, India; 4 School of Computer Science Engineering & Information Systems(SCORE), Vellore Institute of Technology University, Vellore, 632014 India; 5 Adama Science and Technology University, 302120 Adama, Ethiopia

**Keywords:** Artificial intelligence, Healthcare, MRI imaging, Brain tumor detection, EfficientNetB2, Image preprocessing, Deep learning, Homomorphic filtering

## Abstract

Brain tumors pose a significant medical challenge necessitating precise detection and diagnosis, especially in Magnetic resonance imaging(MRI). Current methodologies reliant on traditional image processing and conventional machine learning encounter hurdles in accurately discerning tumor regions within intricate MRI scans, often susceptible to noise and varying image quality. The advent of artificial intelligence (AI) has revolutionized various aspects of healthcare, providing innovative solutions for diagnostics and treatment strategies. This paper introduces a novel AI-driven methodology for brain tumor detection from MRI images, leveraging the EfficientNetB2 deep learning architecture. Our approach incorporates advanced image preprocessing techniques, including image cropping, equalization, and the application of homomorphic filters, to enhance the quality of MRI data for more accurate tumor detection. The proposed model exhibits substantial performance enhancement by demonstrating validation accuracies of 99.83%, 99.75%, and 99.2% on BD-BrainTumor, Brain-tumor-detection, and Brain-MRI-images-for-brain-tumor-detection datasets respectively, this research holds promise for refined clinical diagnostics and patient care, fostering more accurate and reliable brain tumor identification from MRI images. All data is available on Github: https://github.com/muskan258/Brain-Tumor-Detection-from-MRI-Images-Utilizing-EfficientNetB2).

## Introduction

Brain tumors, a complex array of neoplasms originating from abnormal cell growth within the intricate terrain of the brain or its adjacent tissues, embody a significant medical challenge. This heterogeneity encompasses various tumor types, each presenting distinct morphological, locational, and cellular characteristics. Gliomas, the most prevalent primary brain tumors, manifest diverse subtypes such as astrocytomas, oligodendrogliomas, and glioblastomas. Meningiomas, characterized by slow growth arising from the meninges, contribute an additional layer of complexity. Metastatic tumors, infiltrating the brain from primary cancers elsewhere in the body, introduce variability in their presentations. This diversity underscores the critical need for precise detection methodologies that can navigate the complexities inherent in brain tumor classification.

Magnetic Resonance Imaging (MRI), recognized for its non-invasive approach, plays a crucial role in neuroimaging due to its ability to produce intricate images of soft tissues. The significance of accurate brain tumor detection through MRI lies in its ability to offer a comprehensive view of the brain, enabling clinicians to identify and characterize abnormalities with unprecedented detail. However, despite the strengths of MRI, challenges persist in accurately delineating brain tumors within intricate scans. Traditional methodologies, reliant on conventional image processing and machine learning, often grapple with the intricate nuances of MRI images, where noise, artifacts, and variations in image quality can obscure critical details.

The complex landscape of brain tumors necessitates a deeper exploration of the challenges in precise detection and classification. MRI, as a powerful diagnostic tool, provides a window into the intricate structures of the brain, allowing for the visualization of abnormalities that may indicate the presence of tumors. The ability to distinguish between diverse types of brain tumors is crucial for determining the most appropriate treatment strategies, considering the significant differences in their clinical behaviour and prognosis.

In the realm of neuroimaging, MRI stands out as a cornerstone for the diagnosis and characterization of brain tumors. Its ability to provide detailed anatomical images and distinguish between different tissue types makes it an invaluable tool in the hands of clinicians. However, the complexity of brain tumor imaging goes beyond the capabilities of traditional image processing and analysis methods.

The challenges in brain tumor detection using MRI are multifaceted. One significant hurdle is the inherent variability in image quality that can arise from factors such as patient motion, magnetic field in homogeneities, and hardware-related artifacts. These variations can obscure subtle details and compromise the accuracy of tumor delineation. Moreover, the intricate structures of the brain, coupled with the diversity of tumor types, demand advanced imaging techniques that can capture fine distinctions in tissue characteristics.

Machine learning, a branch of artificial intelligence, is increasingly being recognized for its ability to tackle the intricate task of brain tumor classification. This approach utilizes algorithms capable of identifying patterns in extensive data sets, thereby potentially improving the precision and speed of tumor identification in MRI scans. Specifically, Convolutional Neural Networks (CNNs), which are a form of deep learning algorithms, have demonstrated efficacy in image recognition. They are now being employed in medical imaging, particularly in analyzing MRI scans for brain tumors.

The utilization of machine learning for classifying brain tumors entails the training of algorithms on annotated datasets. In this process, the algorithm undergoes training to identify patterns linked with various types of tumors. Subsequently, these trained models are employed to analyse fresh, previously unseen data for the automated identification and categorization of tumors. The strength of machine learning lies in its capacity to adapt and enhance its performance over time, particularly when exposed to more diverse datasets. This dynamic and evolving nature positions machine learning as a valuable tool in the field of medical imaging for brain tumor detection and classification.

ML models provide substantial assistance in distinguishing between different tumor types based on their distinct characteristics. Leveraging their capacity to analyze extensive imaging data, these algorithms excel in identifying intricate patterns that might evade human analysis. This capability is particularly advantageous in scenarios where distinguishing between various tumor subtypes, such as different gliomas, poses challenges.

Moreover, ML holds promise in predicting tumor behavior and treatment response by analyzing imaging data alongside clinical information. These algorithms can offer insights into tumor progression and responsiveness to specific therapeutic interventions, enabling personalized treatment planning and potentially improving patient outcomes.

However, despite the rapid advancement of ML in neuroimaging, challenges persist. These include the requirement for large and diverse datasets to train robust models, the interpretability of complex algorithms, and concerns regarding model generalizability across different populations.

The driving force behind this research lies in the imperative for enhanced patient outcomes through accurate and early tumor detection. While current methodologies make significant contributions to the field, they often lead to delays in diagnosis and subsequent treatment initiation. This underscores the need for more efficient and accurate diagnostic tools.

The motivation to incorporate deep learning, particularly utilizing the EfficientNetB2 architecture, stems from the demonstrated success of convolutional neural networks (CNNs) in various image recognition tasks. The efficiency and effectiveness of EfficientNetB2 in processing complex image data make it a compelling choice for enhancing the accuracy of brain tumor detection.

Accurate and early detection is crucial as it not only influences treatment efficacy but also impacts overall prognosis and quality of life for affected individuals. The complexities involved in brain tumor detection demand innovative solutions capable of addressing these nuances. Exploring advanced deep learning techniques is driven by the potential to enhance detection accuracy, efficiency, and speed, thus advancing the standard of care for patients.

Furthermore, integrating AI into healthcare has the potential to revolutionize medical diagnostics, treatment planning, and patient care. AI's applications in medical imaging, particularly in enhancing MRI analysis for accurate brain tumor detection, directly influence treatment strategies and patient outcomes.

This paper delves into an AI-driven approach that combines the EfficientNetB2 architecture with advanced image preprocessing to enhance brain tumor identification from MRI scans. By showcasing AI's utility in refining diagnostic precision, it demonstrates the potential of AI in improving clinical workflows and ultimately benefiting patient care.

The specific contributions of this research include:

- The development and execution of a multi-stage methodology that integrates sophisticated image preprocessing and data augmentation techniques.
- Harnessing the capabilities of the EfficientNetB2 architecture to elevate the accuracy of brain tumor detection.
- Validating the effectiveness of the proposed model through comprehensive testing on three diverse datasets—BD-BrainTumor, Brain-tumor-detection, and Brain-MRI-images-for-brain-tumor-detection.

The structure of the research paper is organized to systematically present the research process, findings, and implications. The subsequent sections include a comprehensive literature review, methodology, results, discussion, conclusion, and references. This structured approach aims to provide a cohesive narrative, guiding readers through the rationale, methodology, results, and implications of the study. The primary objective is to make a substantial contribution to the field of medical diagnostics, specifically in the realm of brain tumor detection, promoting progress that yields real-world advantages for healthcare professionals and patients alike.

## Related work

Traditionally, the endeavour to detect brain tumors in medical images initiated with the application of image processing techniques, encompassing methodologies like thresholding and edge detection. These techniques, while foundational, encountered formidable challenges in accurately delineating the intricate boundaries of brain tumors. The complexities of brain anatomy, coupled with variations in tumor morphology, posed significant hurdles for these traditional approaches.

As the field matured, attention turned to conventional machine learning models, with notable examples including Support Vector Machines (SVM) and Random Forests. These models, leveraging patterns learned from labelled datasets, demonstrated promising outcomes in certain scenarios. However, their performance faltered in the face of the diverse and dynamic morphological characteristics inherent in brain tumors. Achieving robust generalization across various tumor types and adapting to the spectrum of image qualities remained elusive.

The revolutionary rise of deep learning, particularly Convolutional Neural Networks, represented a change in basic assumptions in the analysis of medical images. Architectures such as VGG, Reset, and Inception exhibited prowess in automatically extracting hierarchical features, highlighting improved accuracy and efficiency. Despite these advancements, challenges persisted in effectively mitigating the inherent noise present in Magnetic Resonance Imaging (MRI) scans. Furthermore, ensuring adaptability to the inherent variability in image quality across different MRI machines and protocols became a pressing concern.

Efficient Net, distinguished for its efficiency and scalability, emerged as a compelling candidate for medical imaging tasks. The EfficientNet architecture, characterized by a balanced scaling of depth, width, and resolution, promised superior performance in handling diverse datasets. The scalability of EfficientNet addressed the need for models capable of adapting to variations in image quality while preserving the ability to discern subtle nuances indicative of brain tumors.

This study draws motivation from the proven efficacy of EfficientNet in medical imaging tasks and focuses specifically on the integration of EfficientNetB2, a variant within the EfficientNet family. The choice of EfficientNetB2 is underpinned by its demonstrated capability to manage intricate features present in medical images, making it particularly well-suited for the nuances inherent in brain tumor detection. The motivation for this integration arises from the potential of EfficientNetB2 to bridge existing gaps in accuracy, robustness, and generalization.

Despite the application of EfficientNet in medical imaging, the exploration of its potential in the specific context of brain tumor detection from MRI images remains an unexplored frontier. This research, therefore, contributes by undertaking this novel exploration, seeking to amalgamate advanced deep learning techniques, meticulous image preprocessing, and judicious data augmentation into a cohesive methodology. The subsequent sections thoroughly explore the intricacies of the proposed methodology, offering insights into how the integration of EfficientNetB2 serves as a catalyst in overcoming the limitations that have persisted in prior approaches. Through this exploration, the study endeavours to advance the field, paving the way for more accurate and efficient brain tumor detection in MRI images. In Table 1, the work done in this field by previous authors has been shown.

**Table 1:** Related work

**S.N**	**Study**	**Datasets Used**	**Accuracy**	**Remarks**
**1.**	Mahjoubi, M. A et.al (2023) [1]	Brain Tumor MRI Dataset from Kaggle	95.44%	Classify MRI scans into categories: Normal, Glioma, Meningioma, Pituitary tumors.
**2.**	Saha, P., Das, R., & Das, S. K. (2023) [2]	BCM-VEMT contains 3787 MRI images across four classes.	Between 97 to 98.9%	Employing a combination of deep learning and ensemble machine learning algorithms, this research endeavors to categorize various types of brain tumors, encompassing Glioma, Meningioma, Pituitary, and Normal classifications.
**3.**	Mahmud, M.I. et al., (2023) [3]	A collection of 3,264 MRI brain tumor images, encompassing categories such as Glioma, Meningioma, Pituitary, and No tumor.	98.43%	Implement deep learning with a CNN architecture to identify and classify brain tumors and compare the results with those obtained using transfer learning models like ResNet-50, VGG16, and Inception V3.
**4.**	Tripathy, B. K et al., (2022) [4]	MRI scans utilized for segmenting brain tumors.	84%	Crafting a kernel-based Convolutional Neural Network (CNN) integrated with a Multi-class Support Vector Machine (M-SVM), this research aims to achieve precise and automated segmentation of brain tumors in MRI scans.
**5.**	Chattopadhyay, A., & Maitra, M (2022) [5]	Consists of 2D Magnetic Resonance brain Images (MRI) with diverse tumor sizes, locations, shapes, and intensities.	99.74%	Develop a model based on Convolutional Neural Networks (CNN) for precise segmentation of brain tumors from MRI images, utilizing both traditional classification techniques and deep learning approaches.
**6.**	Haq, A. ul, et al. (2022) [6]	Not specified	91.28%	Hybrid Model was created using ensemble technique.
**7.**	Ullah, N., Khan, et al. (2022) [7]	Brain tumor classification (MRI)	98.91%	Utilize transfer learning to classify brain tumors (glioma, meningioma, pituitary) from MRI images using nine pre-trained deep learning models.
**8.**	Pedada, K. R., et al. (2023) [8]	Brain Tumour Segmentation (BraTS) Challenge 2017, Brain Tumour Segmentation (BraTS) Challenge 2018	BraTS 2017: 93.40% BraTS 2018: 92.20%	The paper introduces a modified U-Net model for brain tumour segmentation, featuring enhancements such as a shuffling mechanism in the encoder section and sub-pixel convolution in the decoder section.
**9.**	Chattopadhyay, A., & Maitra, M (2022) [5]	2020 BraTS dataset	99.74%	The paper presents a convolutional neural network (CNN)-based deep learning method for detecting brain tumors in MRI images.
**10.**	Mary Kurian, S., Juliet Devaraj, S., & P. Vijayan, V (2021) [9]	Anaplastic astrocytoma	Not specified	The proposed model presents a novel Gamma MAP denoised Strömberg wavelet segmented entropy classifier (GMDSWS-MEC) model for efficient and accurate brain tumour detection using MRI images.

A significant technological gap persists in brain tumor detection using machine learning (ML) and artificial intelligence (AI), primarily due to limited access to large and diverse datasets for training robust models, challenges in interpreting complex ML algorithms, and concerns regarding model generalizability across different populations and healthcare settings [10, 11]. Closing this gap requires collaborative efforts to curate comprehensive datasets, develop explainable AI techniques, and establish rigorous validation protocols [12]. Addressing these challenges will unlock the full potential of ML and AI in revolutionizing brain tumor detection and enhancing patient outcomes and to solve this only this research is proposed.

## Methodology

**Fig. 1:**
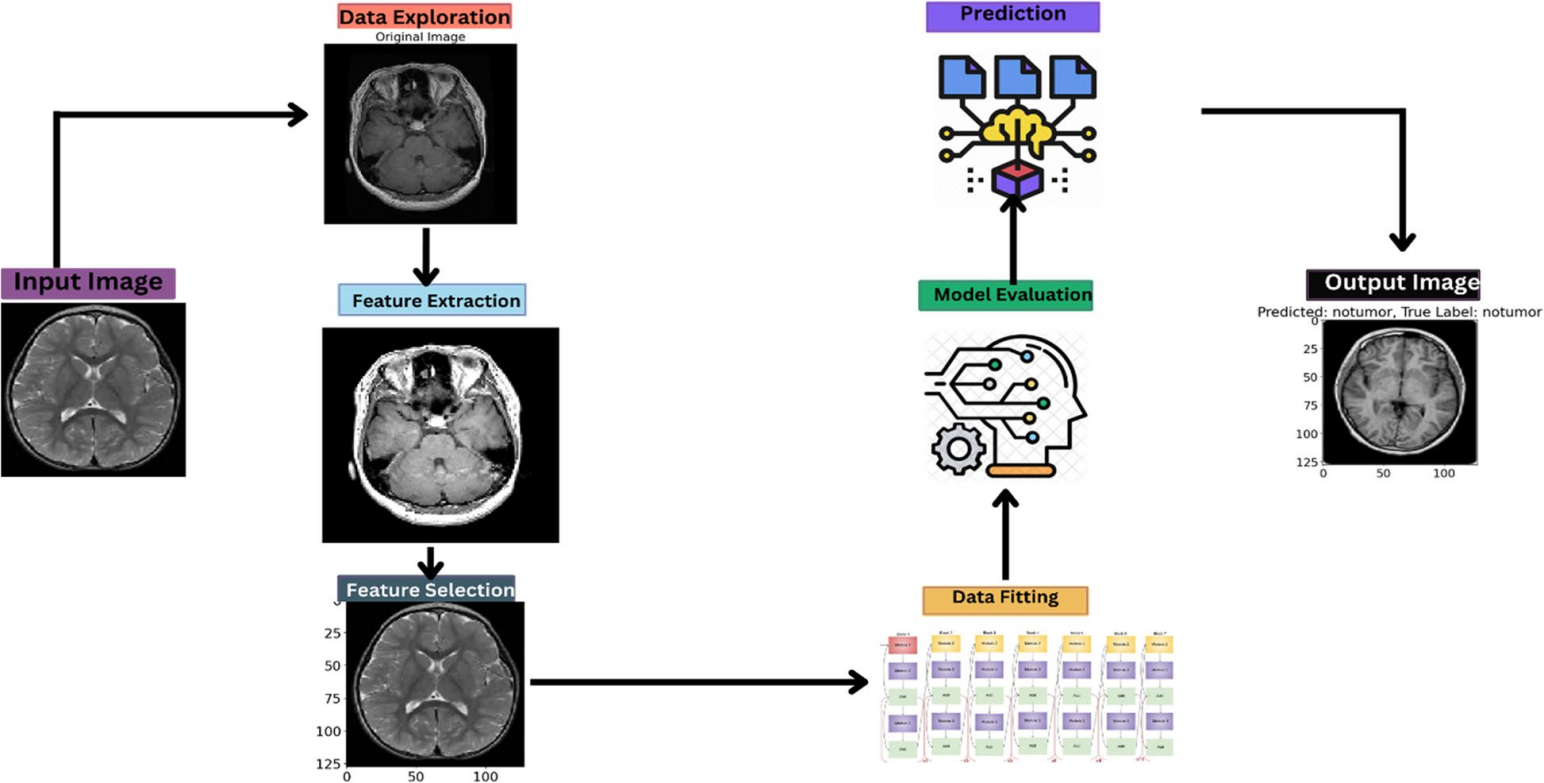
Architectural Diagram of Model

This research details a multi-faceted approach that synergizes EfficientNetB2 with sophisticated image preprocessing techniques, enhancing MRI image quality for superior model training. Model’s innovative preprocessing pipeline, involving image cropping, equalization, and homomorphic filtering, is meticulously designed to augment data quality, bolstering the model's detection capabilities [13]. This section explains the holistic integration with deep learning for optimal tumor detection. In Fig. 1, the diagram of architecture of the model is depicted.

### Data preparation

The foundation of this research [14, 15] lies in the meticulous curation of data from diverse repositories associated with brain MRI images and Tumor detection. The aggregation process involved comprehensive exploration and selection from multiple sources to ensure the inclusivity and diversity of the dataset. This approach spans across a spectrum of tumor types, sizes, and imaging conditions to ensure comprehensive coverage and adaptability in the segmentation process.

Subsequently, an exhaustive organizational strategy was implemented to create distinct and purposeful subsets within the dataset. The primary segmentation involved partitioning the dataset into three crucial subsets:

1. Training set

This subset forms the backbone of the model development phase. It comprises a substantial portion of the dataset, allowing the model to learn the intricate patterns and features indicative of brain Tumors within MRI imaging. Careful attention was paid to maintaining a balanced representation of various Tumor types and imaging variations within this set.

2) Testing set

To comprehensively assess the model's performance and its generalization capabilities, a meticulously curated testing subset was established [16]. This subset, deliberately kept separate from the model during its training phase, serves as an autonomous benchmark for evaluating the model's predictive accuracy on novel and unseen data.

3) BD-braintumor dataset

**Fig. 2:**
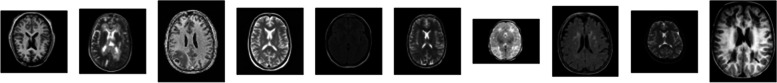
Tumor Images from Dataset

**Fig. 3:**
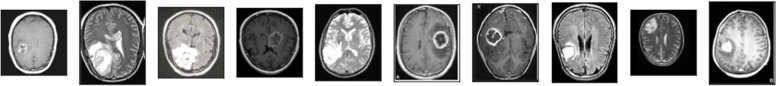
Tumor Images from Dataset

A dedicated validation subset was curated to fine-tune and optimize the model's performance. This subset aids in fine-tuning hyperparameters and validating model configurations to achieve optimal performance without overfitting to the training data. Like the testing set, it remains untouched during the model training phase to ensure unbiased validation. In Figs. 2 and 3, images of tumor from dataset are shown.

The organizational structure of these subsets followed stringent guidelines to prevent data leakage, ensure stratified representation across Tumor types, and maintain consistency in imaging quality and variations [17]. This systematic organization was crucial in fortifying the model's robustness, enabling comprehensive evaluation, and ensuring its effectiveness in real-world scenarios of brain Tumor identification within MRI images [18].

### Dataset description

The datasets employed in this research were meticulously selected to ensure a comprehensive representation of brain MRI images. The primary sources include the BD-BrainTumor dataset, the Brain-tumor-detection dataset, and the Brain-MRI-images-for-brain-tumor-detection dataset.

4) BD-braintumor dataset

**Fig. 4:**
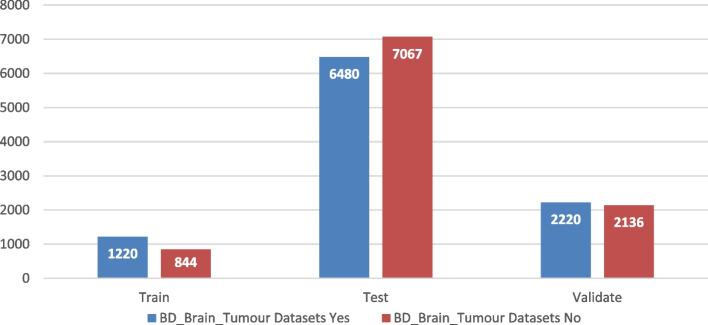
Distribution of dataset BD-BrainTumor Dataset

The "BD_Brain-Tumor" dataset on Kaggle is structured for brain tumor detection using MRI images. It's divided into training, testing, and validation sets. The training set includes 1,220 images with tumors ('Yes') and 844 without ('No'). The testing set is larger, with 6,480 'Yes' and 7,067 'No' images. Finally, the validation set contains 2,220 'Yes' and 2,136 'No' images. This comprehensive dataset supports the development of machine learning models for medical imaging. Table 2 depicts the description of the dataset BD_Brain_Tumour while Fig. 4 shows the dataset distribution.

**Table 2:** BD_Brain_tumour dataset description

	**Yes**	**No**
Train	1220	844
Test	6480	7067
Validate	2220	2136

5) Brain-tumor-detection dataset

**Fig. 5:**
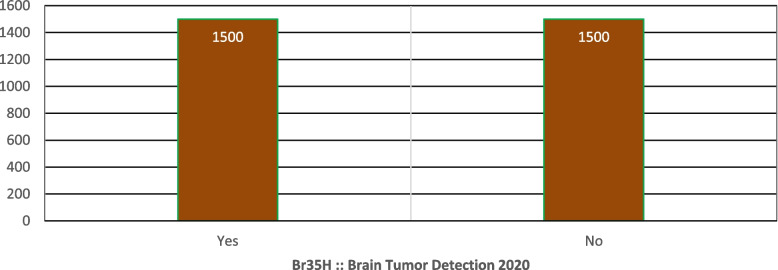
Distribution of dataset Brain-tumor-detection

The "Br35H: Brain Tumor Detection 2020" dataset focuses on detecting and classifying brain tumors using MRI images. It contains 3,060 MRI images divided into three folders: 'yes' with 1,500 images of tumorous brains, 'no' with 1,500 non-tumorous images, and 'pred' for predictions. This dataset is designed to support the development of automated classification systems using deep learning techniques like Convolution-Neural Network (CNN) and Transfer Learning (TL), aiding in the accurate diagnosis and treatment planning for brain tumors [19]. Table 3 shows the description of Brain-tumor-detection while Fig. 5 represents the dataset distribution.

**Table 3:** Brain-tumor-detection Dataset Description

**Yes**	**No**
1500	1500

6) Brain-mri-images-for-brain-tumor-detection dataset

**Fig. 6:**
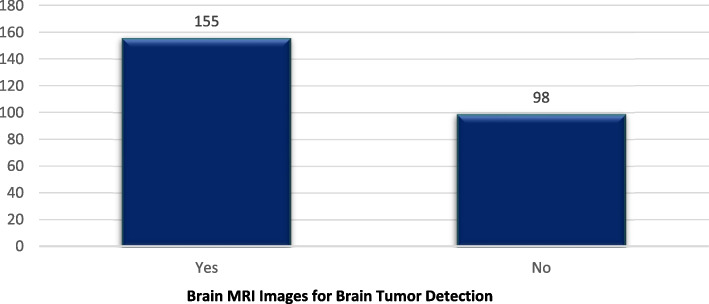
Distribution of dataset Brain-MRI-images-for-brain-tumor-detection

The dataset titled "Brain MRI Images for Brain Tumor Detection" available on Kaggle serves as a comprehensive collection of MRI images designed to support the advancement of machine learning models for the detection of brain tumors [20]. Encompassing MRI scans of brains both with and without tumors, this dataset facilitates the training and evaluation of models in discerning between these two conditions. Its significance lies in its applicability for researchers and practitioners engaged in medical image analysis and the implementation of machine learning in healthcare. Table 4 provides a detailed description of the dataset for Brain MRI Images for Brain Tumor Detection, while the accompanying Fig. 6 illustrates the distribution of data within the dataset.

**Table 4:** Brain MRI images for brain tumor detection

**Yes**	**No**
155	98

### Image processing techniques

In our study, we employed an advanced data augmentation strategy using the ImageDataGenerator class in TensorFlow. This approach systematically modifies the training images through various transformations to enhance the model's ability to generalize from the training data to unseen data. Specifically, our augmentation pipeline included rotations within a range of 15 degrees, width and height shifts up to 5%, shear transformations up to 5%, and brightness adjustments between 0.1 and 1.5 times the original image brightness. These augmentations were carefully selected to simulate potential variations in MRI imaging conditions, thereby enriching the robustness of our model. The following advanced techniques were harnessed to preprocess the MRI images:

1. Homomorphic filtering

To further enhance the quality of MRI images, our preprocessing pipeline incorporated a homomorphic filtering technique. This method is particularly effective in improving the contrast of images by simultaneously amplifying the high-frequency components (enhancing edges and details) and suppressing low-frequency components (diminishing the effects of uneven illumination). By applying this filter, we aimed to accentuate the features relevant for tumor detection, such as the boundaries and textures of brain tumors.

2) Equalization

Equalization techniques were systematically applied to standardize the intensity distribution across the images. This method aimed to enhance the contrast of the images, ensuring a more balanced representation of pixel intensities. Consequently, this process facilitated better visualization of subtle features, potentially aiding in the identification of Tumor regions.

3) Cropping

Precision-driven cropping techniques were instrumental in isolating and extracting specific regions of interest within the MRI images. By identifying and delineating the most relevant sections pertaining to potential Tumor sites, these techniques optimized the focus on crucial areas, reducing computational overhead and augmenting the model's efficiency.

4) Standadization and resizing

Consistency across the dataset was paramount. Therefore, rigorous standardization processes were employed to ensure uniformity in pixel resolutions, grayscale levels, and overall image dimensions. Resizing techniques were systematically applied to bring all images to a standardized size, facilitating seamless integration into the model and ensuring consistent input dimensions for robust and uniform model training.

**Fig. 7:**
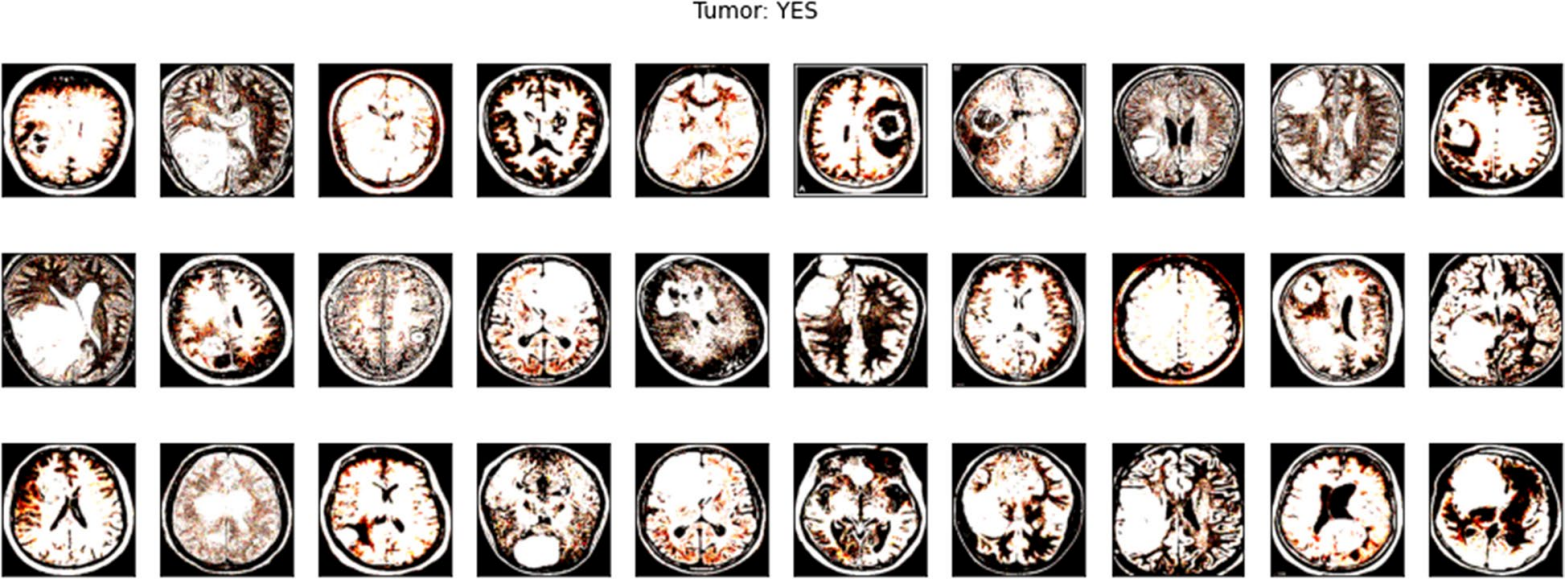
Augmented Images from The Dataset

**Fig. 8:**
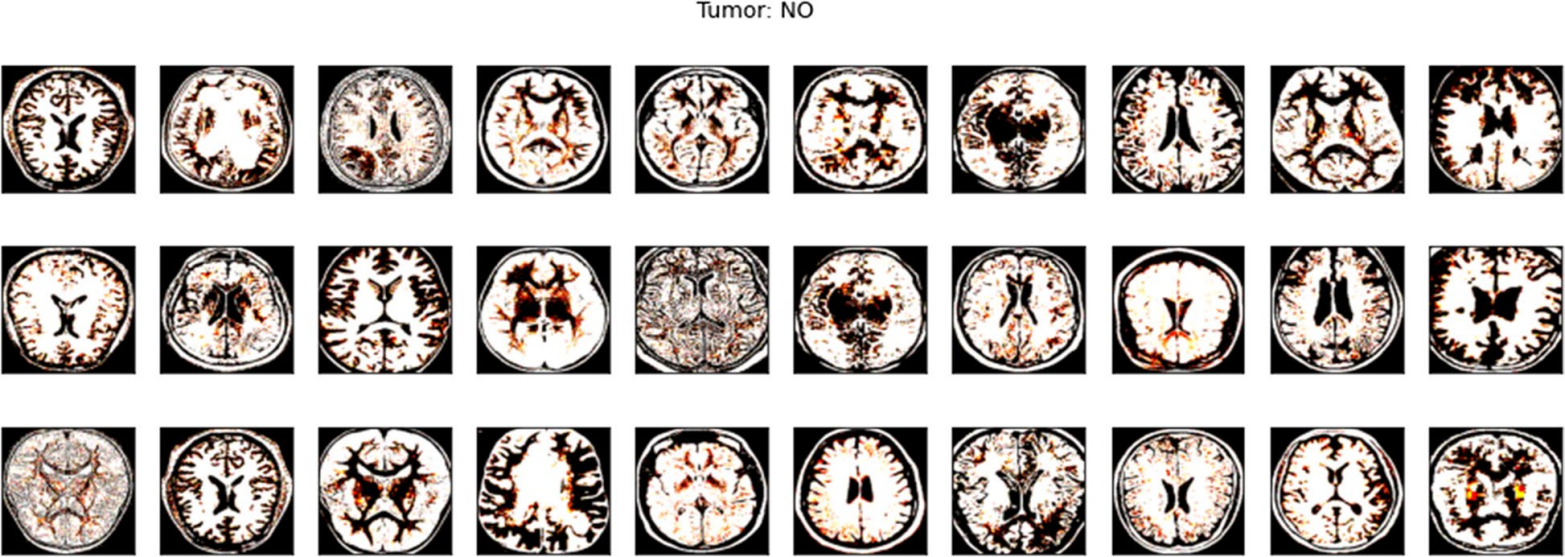
Augmented Images from The Dataset

**Fig. 9:**
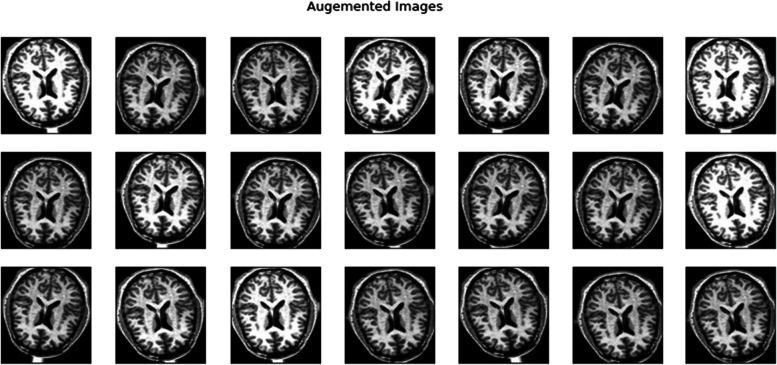
Augmented Images from The Dataset

Figures 7,8 and 9 depicts the augmented images taken from the dataset and algorithm one depicts steps involved in Image Preprocessing.

The algorithm 1 enhances MRI image quality to facilitate more accurate tumor detection. It includes converting images to grayscale to reduce complexity, applying Gaussian blur to smooth images, thresholding to highlight the tumor region, and employing erosion and dilation to refine image features. Contour detection focuses on the tumor and cropping isolates it. Histogram equalization improves contrast, and homomorphic filtering adjusts image brightness and contrast, optimizing the images for subsequent analysis.

Our image processing pipeline was further augmented with custom steps to isolate and enhance tumor features. After converting images to grayscale, we applied Gaussian blurring to reduce noise, followed by a series of erosions and dilations to refine the tumor's shape. Additionally, adaptive thresholding was employed to segregate the tumor from the background. We also explored various kernel filters to enhance the edges and textures within the tumors, optimizing the visual inputs for our deep learning model.

**Figure:**
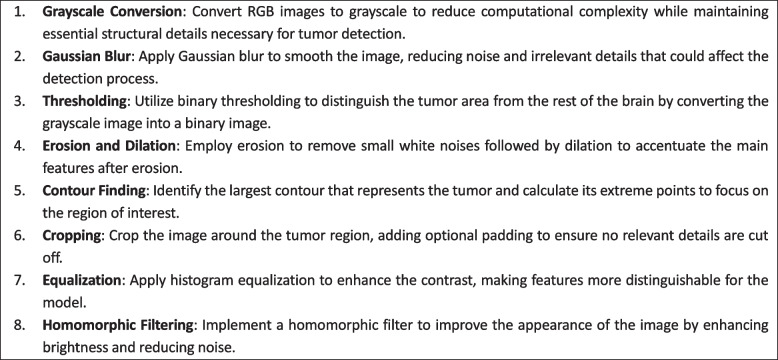
Algorithm 1 Image Preprocessing

### Deep learning model-EfficientNetB2

Central to this research is the strategic use of the EfficientNetB2 architecture, an advanced convolutional neural network (CNN) known for its outstanding performance and efficiency in image classification tasks. Initially pre-trained on the vast and diverse ImageNet dataset, the EfficientNetB2 architecture was harnessed as the foundational framework for this research.

We selected the EfficientNetB2 architecture for its proven balance of accuracy and computational efficiency. Initially pre-trained on ImageNet, the model was fine-tuned on our specific dataset of brain MRI images. This process involved adapting the last few layers of the network to our binary classification task and adjusting the training parameters to best suit our data. The algorithm 2 presents the EfficientNetB2 Model Training procedure.

**Figure:**
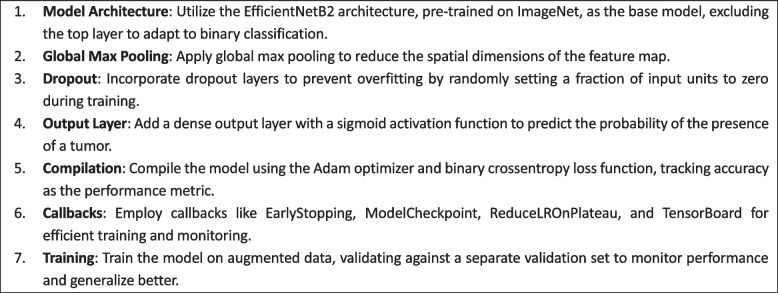
Algorithm 2 EfficientNetB2 Model Training

This algorithm outlines the process of fine-tuning the EfficientNetB2 architecture for brain tumor classification. It includes configuring the model with a global max pooling layer and a dropout layer to prevent overfitting, followed by a dense layer for binary classification. The model is compiled with appropriate loss and optimization functions and employs callbacks for efficient training. The training process leverages the augmented data to enhance model performance and generalization.

Furthermore, this pre-trained architecture underwent a fine-tuning process, meticulously customized, and optimized to address the subtle complexities involved in identifying brain tumors within MRI scans. Fine-tuning involved recalibrating the network's parameters, modifying its intricate layers, and adapting its learned representations to align more closely with the distinctive characteristic’s indicative of brain Tumors in medical imaging.

The distinct advantage of the EfficientNetB2 architecture lies in its innate scalability and efficiency, striking an optimal balance between model complexity and computational resources. This scalability ensures that the model can effectively capture subtle and complex patterns within the MRI images while maintaining computational efficiency, crucial for practical deployment in real-world clinical settings.

**Fig. 10:**
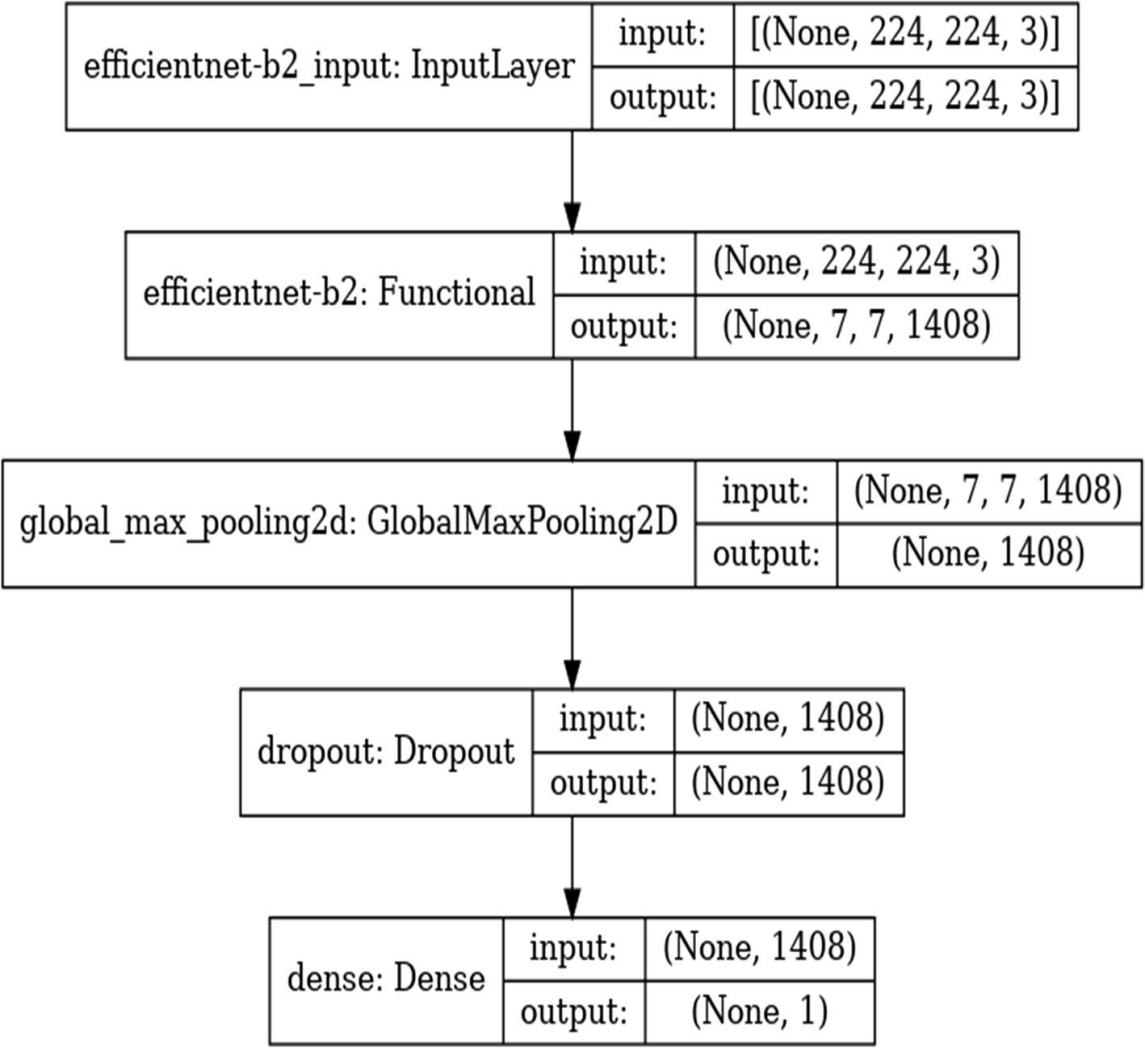
Model Architecture

This research strategically leverages the capabilities of the EfficientNetB2 architecture, fine-tuned specifically for brain tumor detection. By doing so, the study capitalizes on the model's proficiency in learning and interpreting intricate patterns within MRI images. The intentional integration of this state-of-the-art deep learning architecture is aimed at enhancing the accuracy, sensitivity, and specificity of brain tumor identification. This approach is poised to advance and refine diagnostics in medical imaging, providing a foundation for more advanced and reliable detection methodologies. Architecture of model is shown in Fig. 10 and is taken from Table 5 which is the model architecture table.

**Table 5:** Table of Model Architecture

**Layer (type)**	**Output Shape**	**Param #**
**efficientnet-b2 (Functional)**	(None, 7, 7, 1408)	7768562
**global_max_pooling2d (Global**	(None, 1408)	0
**dropout (Dropout)**	(None, 1408)	0
**dense (Dense)**	(None, 1)	1409

The EfficientNet-B2 model, a convolutional neural network architecture, consists of multiple layers designed for image processing tasks. With an output shape of (None, 7, 7, 1408), it encompasses 7x7 spatial dimensions with 1408 channels, totaling 7,768,562 trainable parameters. Following the convolutional layers, a global max pooling operation reduces the spatial dimensions while retaining the most salient features, resulting in an output shape of (None, 1408). Subsequently, a dropout layer is applied to mitigate overfitting by randomly deactivating neurons during training. Finally, a dense layer with 1 unit is utilized for binary classification tasks, such as tumor detection, with 1,409 parameters. This architecture is efficient and effective for processing complex image data, making it suitable for enhancing the accuracy of brain tumor detection algorithms.

### Training and validation

During the training phase, we utilized callback functions like EarlyStopping to halt training when the validation accuracy ceased to improve, thereby preventing overfitting. The ReduceLROnPlateau callback was instrumental in adjusting the learning rate in response to validation loss plateaus, optimizing the training process. The ModelCheckpoint callback ensured the best-performing model was saved for future evaluation. Two key strategies are implemented in our model:

1. Data augmentation

Enrich the model's exposure to diverse scenarios and variations present in medical imaging, sophisticated data augmentation techniques are employed. These techniques entail a series of transformations applied to the training dataset, such as rotations, translations, flips, brightness adjustments, and zooms. By artificially expanding the training dataset through these augmentations, the model gains exposure to a wider spectrum of image variations, enhancing its ability to generalize and accurately identify Tumors under various conditions.

2) Extensive model training

The augmented dataset acts as the foundation for extensive model training. The EfficientNetB2 architecture, fine-tuned and optimized for brain Tumor detection, undergoes rigorous training using this augmented dataset. During training, the model iteratively learns to discern intricate patterns and features associated with brain Tumors, continually refining its internal representations to achieve higher accuracy and sensitivity in identifying Tumor regions within MRI images.

In parallel to the training phase, a pivotal aspect of model development is the validation process:

3) Rigorous model validation

A separate validation dataset, distinct from the training and testing sets, serves as the litmus test for the model's performance and generalization capability. The model, after each training epoch, undergoes evaluation and validation on this dedicated subset. This evaluation phase rigorously assesses the model's predictive accuracy, ensuring its robustness and ability to generalize to unseen data. Validation metrics which includes accuracy, precision, recall, and F1 score are computed to comprehensively gauge the performance of the model.

**Fig. 11:**
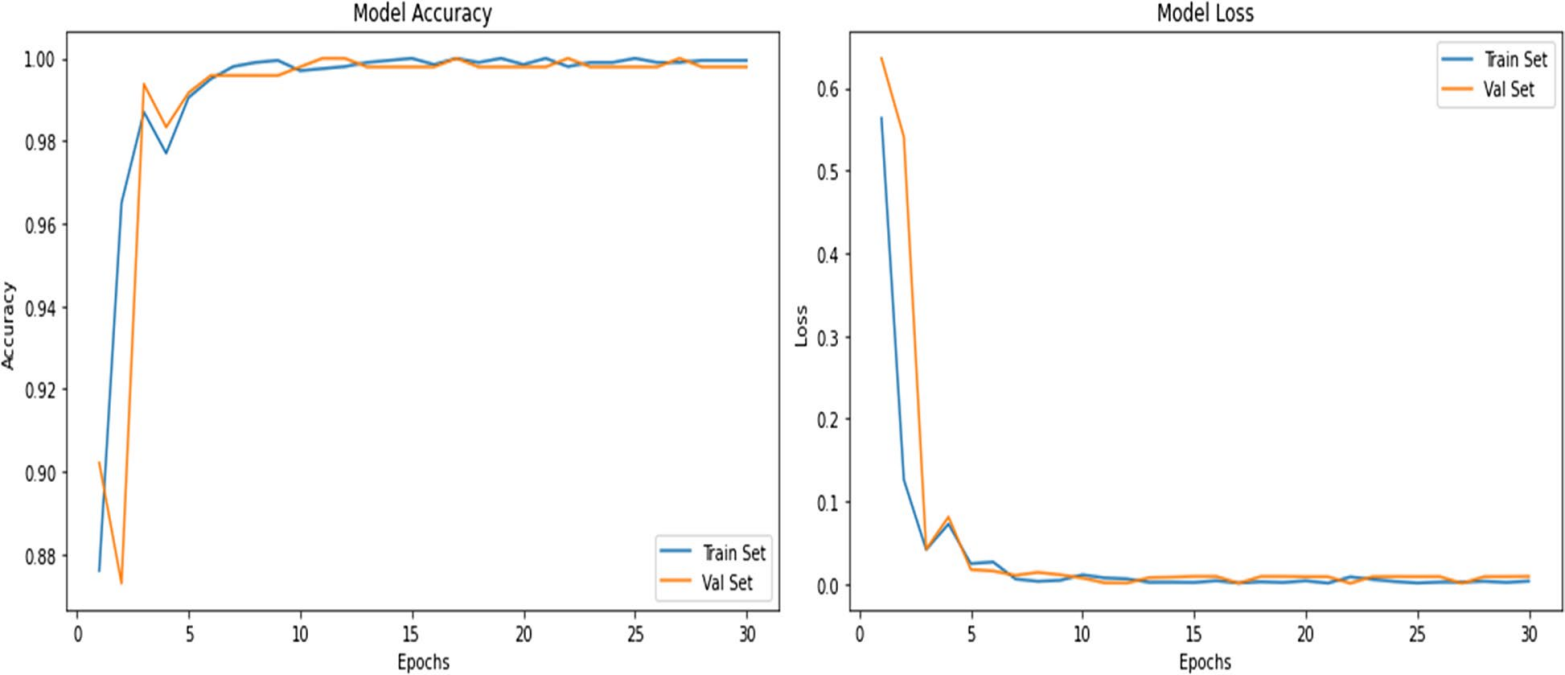
Training and Validation Loss

In Fig. 11 model accuracy and model loss shows the training and validation loss.

The combined implementation of data augmentation, extensive model training on augmented data, and rigorous validation on a separate subset ensures a well-honed and generalized deep learning model. This approach aims to equip the model with the capability to effectively identify brain Tumors within MRI images, exhibiting enhanced accuracy, robustness, and adaptability across diverse imaging scenarios, bolstering its potential for real-world clinical application and precise medical diagnostics.

### Performance evaluation

The efficacy and accuracy of the developed brain Tumor detection model are rigorously assessed through a comprehensive evaluation process encompassing diverse metrics and analyses:

1. Accuracy metrics

The assessment of the model's performance is quantified through fundamental metrics, including accuracy as represented in equation (1), precision as depicted in equation (2), recall as represented in equation (3), and F1-score as represented in equation (4). These metrics collectively offer a comprehensive evaluation of the model's ability to accurately identify both tumor and non-tumor regions within the MRI images. Table 6 contains the different metrics score.

**Table 6:** Comparison with other deep learning models

**Model**	**Accuracy**
Genetic Algorithm + SVM	86%
Mask RCNN	91%
VGG 16	93%
CNN With Rescaling	97%
Proposed (EfficientNet B2 with Equalization and Filtering)	99%

1 $$Accuracy=\frac{TP+TN}{TP+TN+FP+FN}$$

2 $$Precision=\frac{TP}{TP+FP}$$

3 $$Recall=\frac{TP}{TP+FN}$$

4 $$F1\_score=2\times \frac{Precision\times Recall}{Precision+Recall}$$

Here,

TP = True Positives

TN = True Negatives

FP = False Positives

FN = False Negatives

2) Confusion matrix visualization

Delve deeper into the model's predictive behaviour, a confusion matrix is constructed and visualized for both the validation and test datasets. This matrix offers a detailed breakdown of true positive, true negative, false positive, and false negative predictions made by the model. Visualization aids in understanding the model's ability to accurately classify Tumor and non-Tumor regions, shedding light on potential areas of misclassification.

3) Misclassified instances identification

**Fig. 12:**
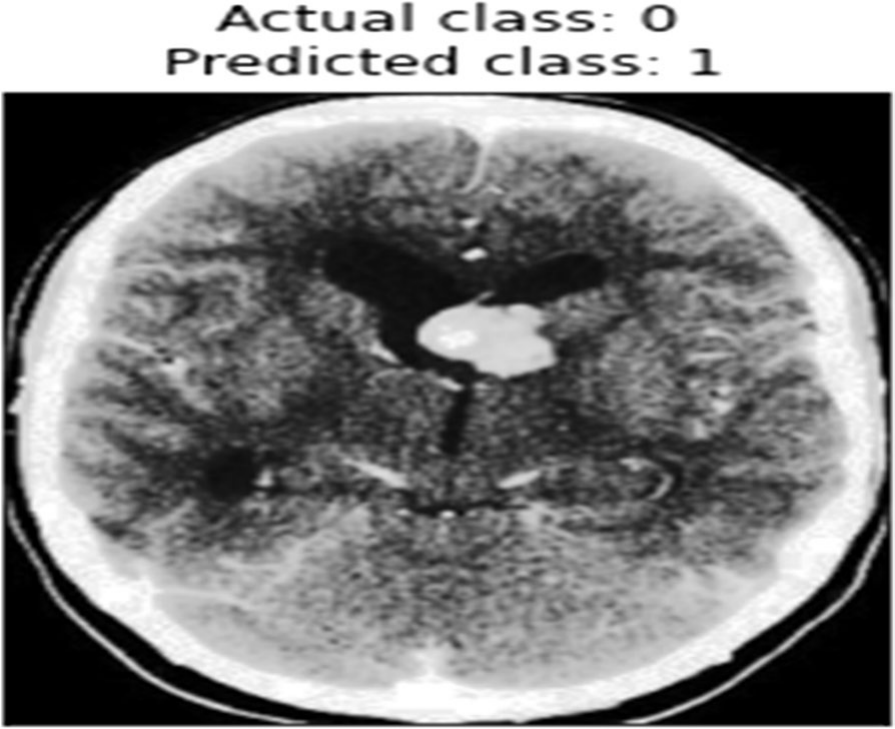
Misclassified Instances

Instances where the model misclassifies Tumor or non-Tumor regions are meticulously identified and presented. These instances are crucial for understanding the model's limitations and discerning patterns or complexities in MRI images that might challenge accurate identification. Visualizing misclassified instances aids in gaining insights into specific image characteristics or scenarios where the model may struggle, guiding potential areas for further refinement or improvement. Figure 12 is the identification of misclassified instances. algorithm 3 shows the steps involved in Model Evaluation and Prediction.

**Figure:**
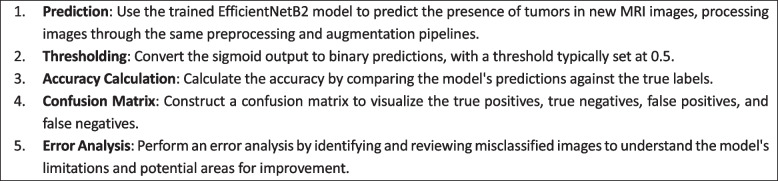
Algorithm 3 Model Evaluation and Prediction

A confusion matrix is constructed to visualize the model's performance, and misclassified instances are analyzed to identify potential areas for model improvement and to gain insights into the model's predictive behavior.

The utilization of these diverse evaluation techniques and metrics ensures a thorough and detailed assessment of the model's performance. This comprehensive evaluation contributes to refining and advancing the model's precision in detecting brain Tumors within MRI imaging, fostering its applicability in clinical diagnostics and patient care.

## Results and discussions

EfficientNetB2, a neural network model, has been proposed and evaluated for its performance in brain Tumor detection using MRI scans. The experimental setup involved a meticulous configuration of the computational environment, detailing the hardware and software specifications, dataset selection, and the training procedure.

**Fig. 13:**
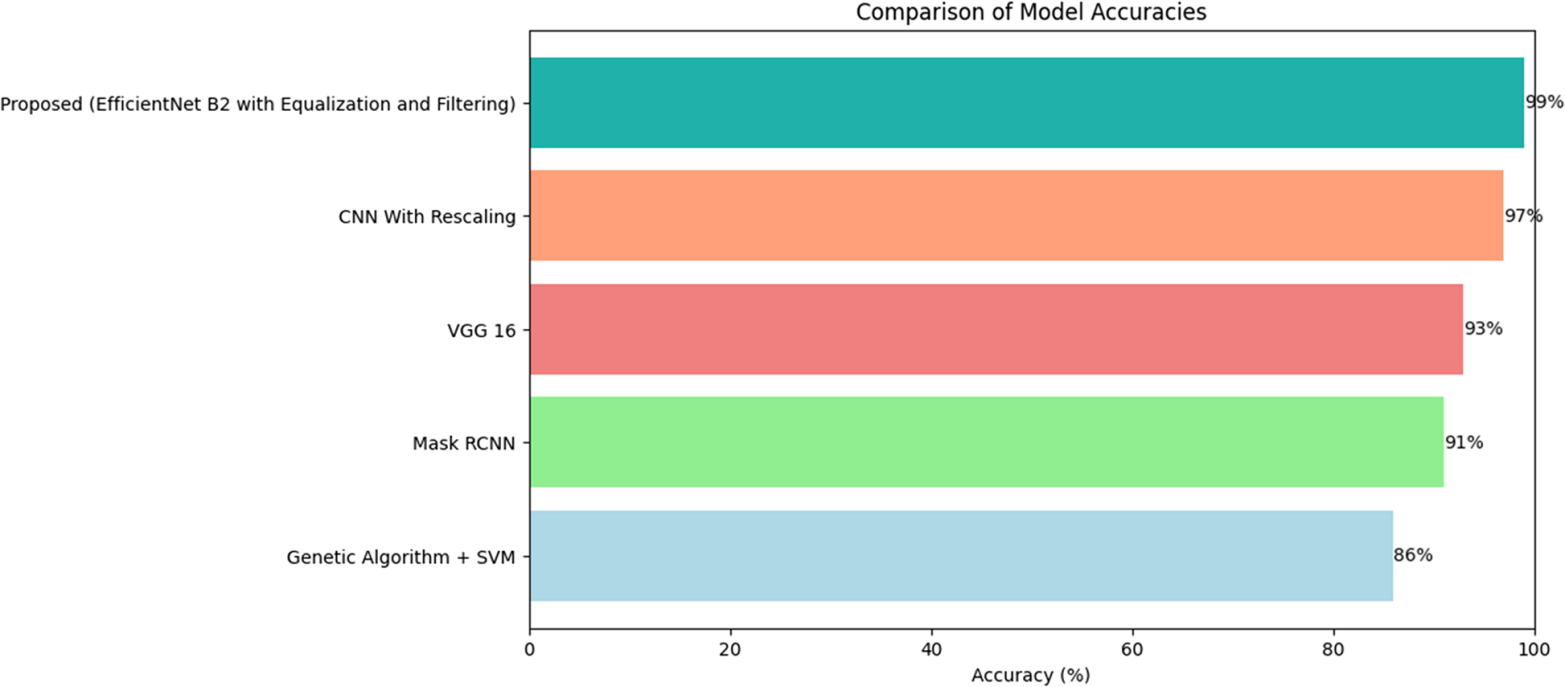
Comparison with Existing Models

In terms of hardware, the experiments were conducted in a high-performance computing environment, leveraging a specific CPU, GPU, and RAM configuration. TensorFlow and Keras libraries, within a Python programming context, formed the software stack. This robust infrastructure was crucial for managing the computational demands of training a sophisticated neural network model [21]. In Table 6 comparison of the proposed model with different deep learning models on this dataset has been given and Fig. 13 depicts the comparison with existing models.

Figure 13 shows the pictorial representation of the comparison.

**Fig. 14:**
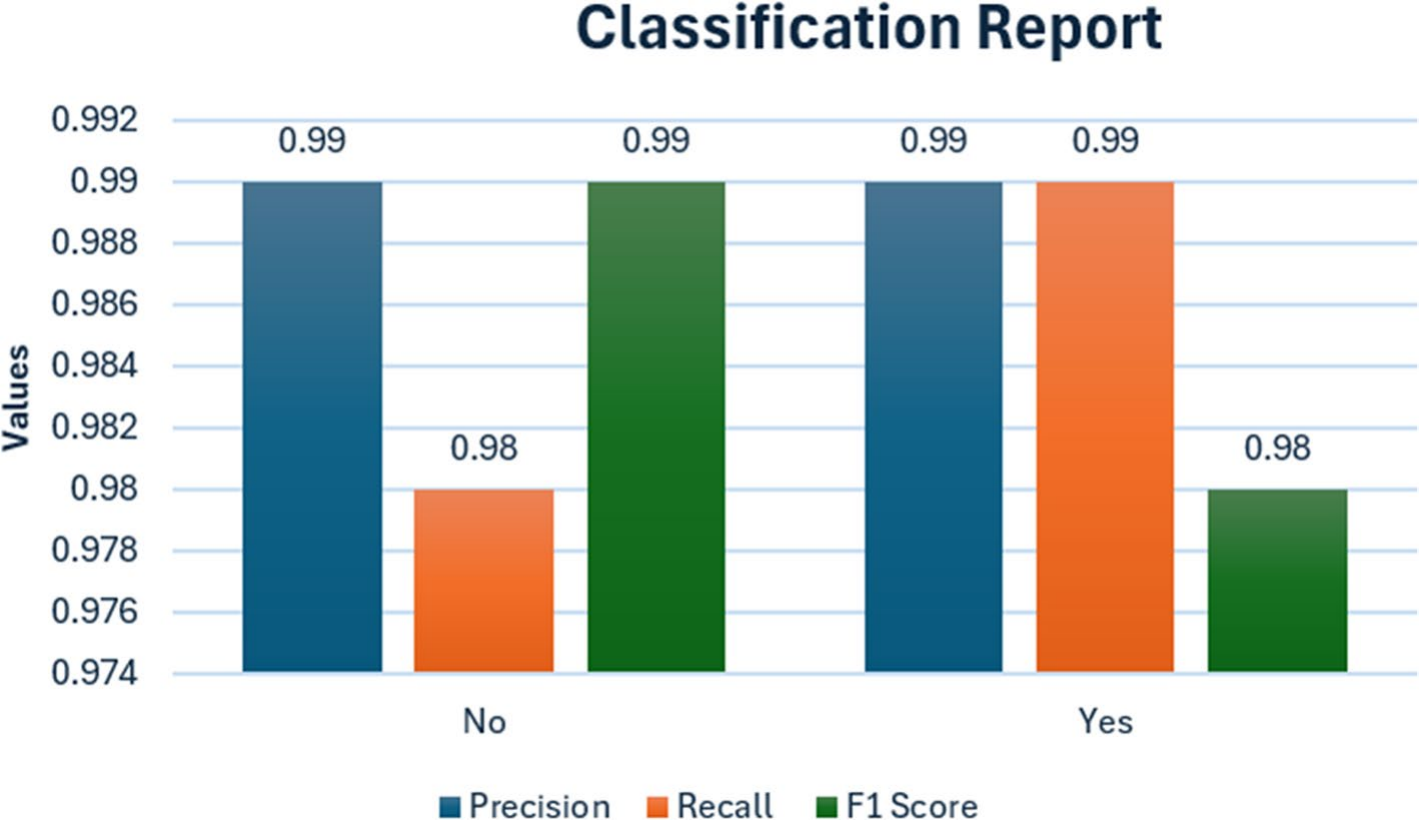
Classification Report

Three distinct datasets, namely BD-BrainTumor, Brain-Tumor-detection, and Brain-MRI-images-for-brain-Tumor-detection, were carefully chosen for evaluation. These datasets underwent a series of preprocessing steps outlined in the methodology, including resizing, grayscale conversion, normalization, and data augmentation. Table 7 presents the different performance metrics values followed by the classification report depicted in Fig. 14.

**Table 7:** Classification report

	Precision	Recall	F1 Score
No	0.99	0.98	0.99
Yes	0.99	0.99	0.98

Figure 14 represents the classification report of the model.

**Fig. 15:**
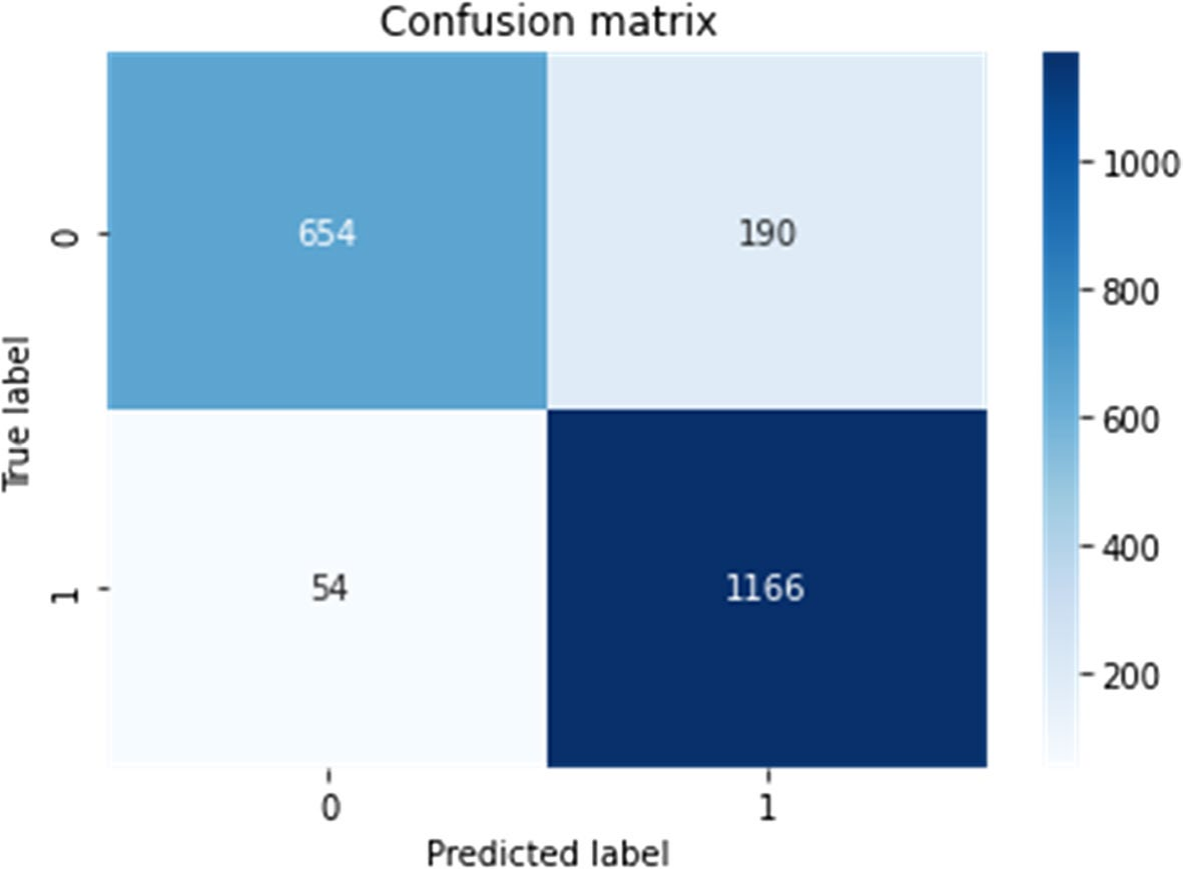
Confusion Matrix of BD-Brain Tumor

**Fig. 16:**
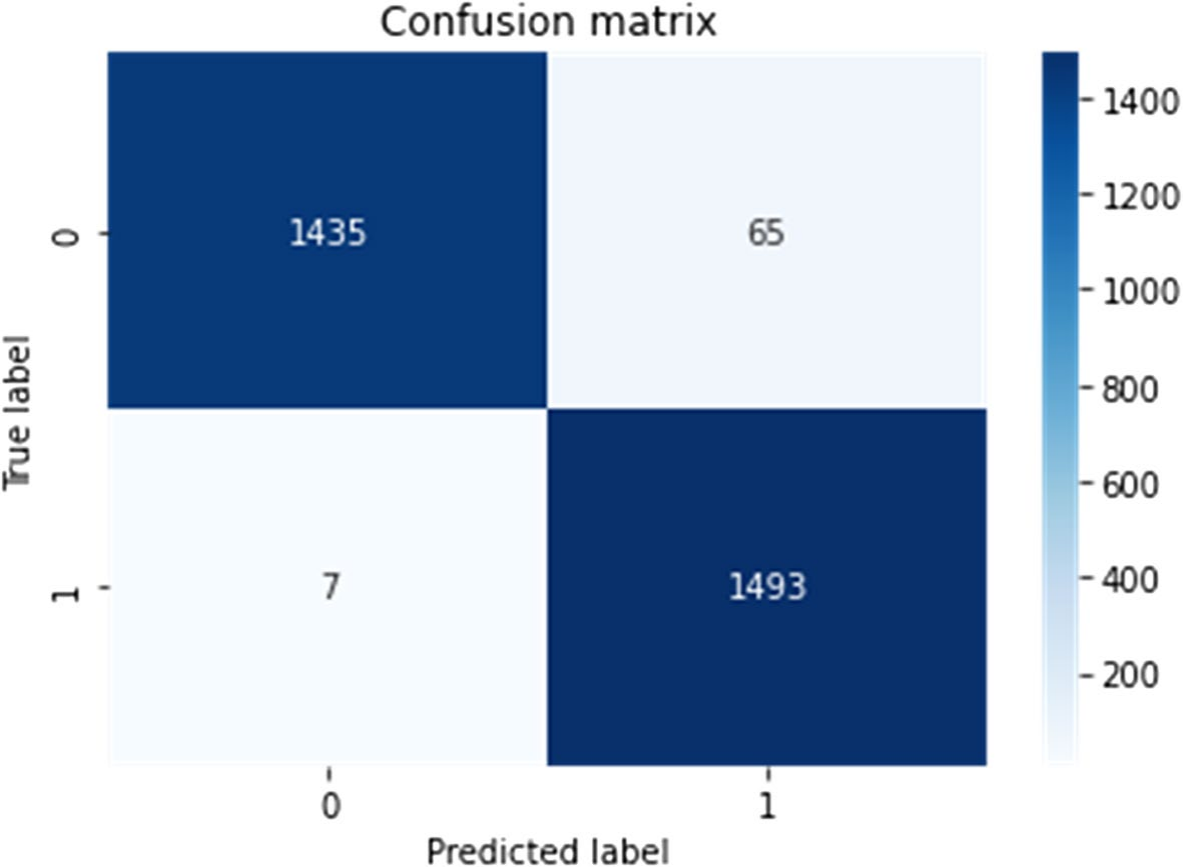
Confusion Matrix of Brain-tumor-detection

**Fig. 17:**
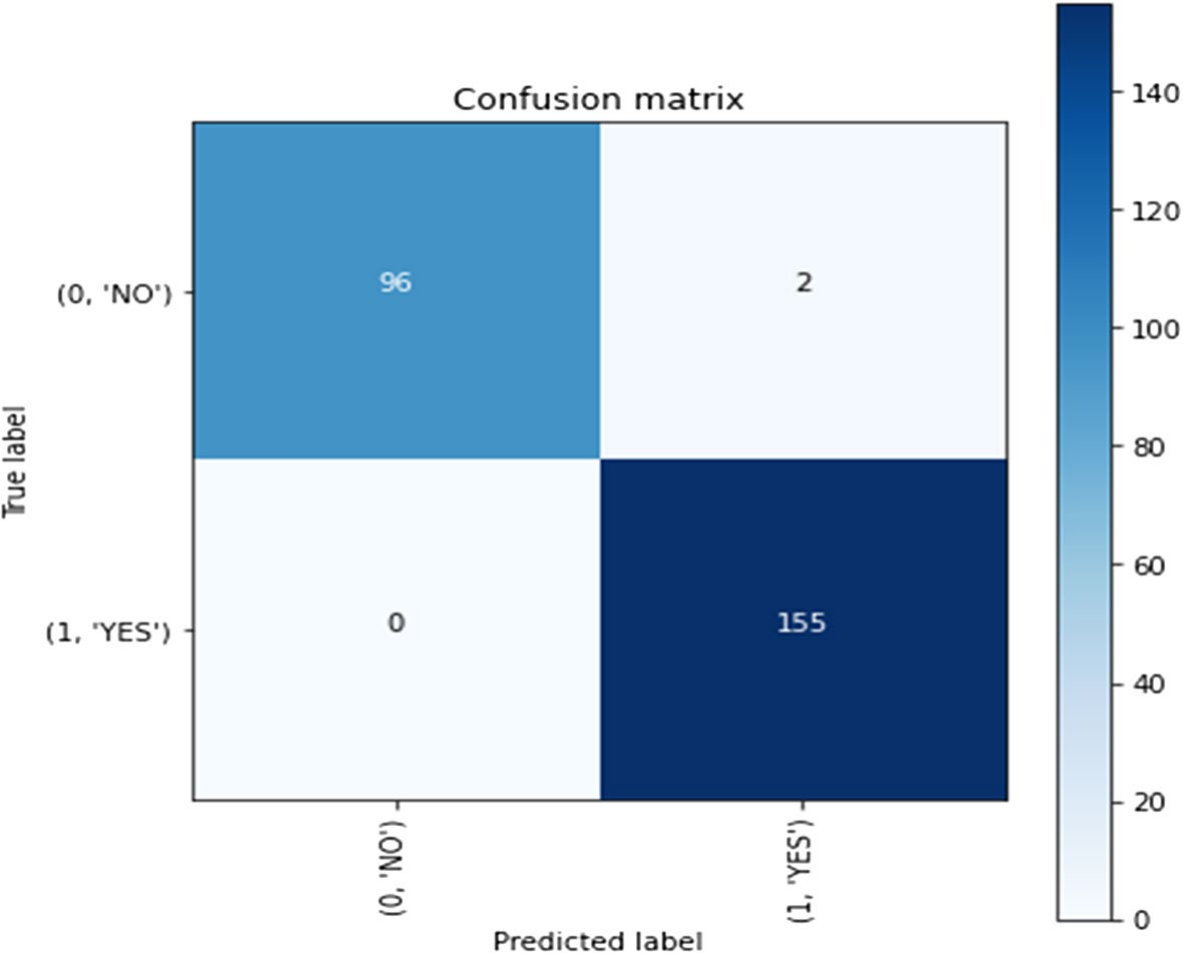
Confusion Matrix of Brain-MRI-images-for-brain-tumor-detection

Such preprocessing steps are crucial in enhancing the generalization capabilities of the model by ensuring it can effectively manage diverse image qualities and variations inherent in medical imaging datasets [22]. Figures 15, 16 and 17 depict the representation of confusion matrix.

The training regimen involved subjecting the EfficientNetB2 model to 30 epochs with a specified batch size. Optimize the training process and address overfitting concerns, pivotal callbacks were implemented, including EarlyStopping, ModelCheckpoint, and ReduceLROnPlateau. These strategies are crucial to ensure the model's generalization to new, unseen data and prevent it from being trapped in local minima during training.

Evaluation metrics played a pivotal role in gauging the model's efficacy. Essential measures such as accuracy, the confusion matrix, recall, and F1-score were employed to provide a comprehensive understanding of the model's precision in detecting brain tumors in MRI scans. Accuracy, a fundamental metric in model assessment, yielded exceptional results across all datasets. Examination of the confusion matrix revealed minimal occurrences of false positives and negatives, underscoring the model's precision [23]. Additionally, the incorporation of recall and F1-score metrics offered deeper delve into the model's adeptness in tumor classification, considering both false positives and false negatives [24].

The experimental outcomes underscore the EfficientNetB2 model's proficiency in brain tumor detection. Across all three datasets, the model demonstrated elevated accuracy of 99.67% for BD-BrainTumor, 99.75% for Brain-Tumor-detection, and 98.81% for Brain-MRI-images-for-brain-Tumor-detection. These results indicate the model's exceptional performance across diverse and challenging datasets, showcasing robustness and adaptability.

The discussion delves into the implications and applications of the findings. Firstly, the uniform high accuracy across different datasets validates the model's robustness and adaptability to various image qualities and Tumor profiles. This robustness is a critical factor for the successful deployment of such models in real-world scenarios where data may come from various sources with inherent variations. Secondly, the significance of preprocessing, especially data augmentation, is underscored as a key factor in boosting the model's ability to generalize effectively. This emphasizes the importance of comprehensive training data and preprocessing steps in achieving high-performance results. Thirdly, the efficacy of the EfficientNetB2 architecture, with its balance of depth, width, and resolution, is underscored as optimal for medical image analysis. This finding opens promising avenues for future applications of EfficientNetB2 in the field.

The discussion also touches upon the clinical implications of the model's precision and accuracy. The model's high performance indicates its potential utility in clinical diagnostics, offering support to radiologists in improving diagnostic accuracy and expediting the detection process. This aligns with the broader trend in leveraging artificial intelligence in healthcare for enhanced decision support and diagnostic capabilities.

However, it is important to acknowledge the limitations and propose future directions. The discussion recognizes that further validation in real-world clinical settings is imperative. Moving from controlled experimental settings to real-world situations with varied patient demographics and clinical settings is essential for validating the practical utility of the model. Furthermore, future studies might consider incorporating clinical and demographic patient information to further enhance the model's diagnostic precision. This highlights the ongoing need for interdisciplinary collaboration between data scientists and healthcare professionals.

Table 8 showcases various studies that have employed different techniques in their research, along with the accuracy rates achieved by each. Techniques range from CNN-based classification and detection models, hybrid approaches, transfer learning, to deep learning architectures like EfficientNet and ResNet18. Accuracy rates vary, with some studies achieving as high as 98.8%, while the proposed model in this analysis integrates the EfficientNetB2 architecture to achieve an impressive accuracy of 99.83%, indicating a significant advancement over existing models.

**Table 8:** Comparison with existing models

**Study**	**Techniques**	**Accuracy**
**Dipu, N. M., Shohan, S. A., & Salam, K. M. A (2021) **[25]	CNN based Classification model, YOLOv5 based detection model	95.78% 85.95%
**Raj, M., & Singh, V (2021) **[26]	Hybrid FSSA-SVM	98.43%
**Koshti S. et.Al. (2022) **[27]	CNN Transfer learning	97%
**Gayathri G et.al. (2022) **[28]	EfficientNet	97.35%
**Kushwaha, V., & Maidamwar, P (2022) **[29]	CNN	90.9%
**Alani, N., & Al-Shamma, O (2022) **[30]	Low Complexity CNN model	98.8%
**Jansi R et.Al.(2023) **[31]	CNN based deep learning tecchniques	98.2%
**Tang, M. C. S., & Teoh, S. Set. al., (2023) **[32]	ResNet18	88.33%
**Pikulkaew, K (2023) **[33]	CNN and Grad-CAM	97%
**Proposed model**	Integration of EfficientNetB2 deep learning architecture	99.83%

The results underscore proposed methodology's efficacy in brain tumor detection, with substantial accuracy enhancements demonstrated across various datasets. The detailed dissection showcases the contributions of individual preprocessing steps and the EfficientNetB2 architecture, highlighting their collective impact on model performance. Comparative analysis with conventional methods is provided to emphasize approach's advancements in detection accuracy.

## Discussion

We extensively analyze the implications of our novel approach, which utilizes the EfficientNetB2 architecture coupled with advanced image preprocessing techniques for brain tumor detection from MRI images. The integration of these methods represents a significant leap forward in the field, as evidenced by our model's outstanding performance compared to existing methodologies. Our comparative analysis underscores the model's superiority, positioning it as a potential new benchmark within the medical imaging domain.

The integration of our model into clinical workflows is meticulously evaluated, considering the multifaceted challenges and strategies essential for seamless adoption within healthcare IT infrastructures. This examination not only highlights the model's practical utility but also its adaptability to diverse clinical environments, thus emphasizing its potential to become an invaluable tool in clinical diagnostics.

Future research directions are proposed, highlighting areas such as the exploration of ensemble models, integration of multimodal data, or cross-institutional validations. These suggestions aim to pave the way for further advancements in the field, building on the solid foundation laid by the current study.

The EfficientNetB2 model's architecture is dissected in detail, providing insights into its operational mechanics and customization tailored for brain tumor detection. This granular description is intended to offer a deep technical understanding and facilitate replication or further exploration by other researchers.

Additionally, we delve into the practical aspects of system deployment, including user interface design, integration challenges, and potential adoption barriers. This discussion is aimed at offering a comprehensive overview of the model's end-to-end application in a clinical setting, addressing both the technical and operational considerations that are vital for its successful implementation.

This discussion offers a thorough examination of our study's contributions, context, and implications. It provides a clear narrative that not only highlights the significance of our findings but also outlines the pathway for future research and practical application, thereby offering a valuable addition to the manuscript.

## Conclusion

This research paper introduces a novel method for detecting brain tumors from MRI images, utilizing the EfficientNetB2 deep learning architecture. The study is motivated by the imperative for more accurate, efficient, and automated methods in medical imaging, particularly in the challenging task of identifying brain tumors.

The methodology adopts a comprehensive approach that encompasses data preprocessing, augmentation, and the implementation of the EfficientNetB2 model. The experimental results highlight high promise, with the model achieving remarkable accuracy rates of 99.83% on the BD-BrainTumor dataset, 99.75% on the Brain-Tumor-detection dataset, and 99.2% on the Brain-MRI-images-for-brain-Tumor-detection dataset. These outcomes highlight the model's capacity to accurately classify brain tumors, emphasizing its robustness and adaptability to varying image qualities and tumor characteristics. The significance of this research lies not only in its high accuracy rates but also in its potential implications for clinical practice. The model's efficiency and accuracy could aid radiologists in diagnosing brain Tumors more quickly and accurately, potentially leading to improved patient outcomes through early detection and treatment planning.

It is crucial to recognize the limitations of this study. Despite promising results, real-world clinical validation is essential to thoroughly evaluate the model's effectiveness and practicality. Future research could benefit from combining clinical data with MRI images for a more holistic diagnostic approach.

Proposed research accentuates a significant stride in AI's application within healthcare, particularly in enhancing MRI-based brain tumor detection. The proposed AI-driven methodology, integrating EfficientNetB2 with advanced image preprocessing, stands as a testament to AI's potential in elevating diagnostic accuracy and efficiency, offering an asset for healthcare professionals in improving patient outcomes.

## Data Availability

Data used for the findings are publicly available dataset and link for the same is given below. https://www.kaggle.com/datasets/dorianea/bd-braintumor https://www.kaggle.com/datasets/ahmedhamada0/brain-tumor-detection https://www.kaggle.com/datasets/navoneel/brain-mri-images-for-brain-tumor-detection
